# Supplementary material for: Characterization of a novel subfamily 1.4 lipase from Bacillus licheniformis IBRL-CHS2: Cloning and expression optimization
Source: PLoS One. 2024 Dec 17;19(12):e0314556. doi: 10.1371/journal.pone.0314556 (PMC11651597; doi:10.1371/journal.pone.0314556)
Supplement: S1 Table — (PDF) [file pone.0314556.s001.pdf]

S1 Table: List of forward and reverse primers

Name	Forward/ Reverse	Sequence (5’ to 3’)	Restriction site
BLF	Forward	ATG CGT CGT CAT TCA TTT TTA	-
BLR	Reverse	TTA TTT CCC GCT GGC GGT CAG	-
BLEF	Forward	CGC CGG CAT ATG CGT CGT CAT TCA TTT TTA	*Nde*I
BLER	Reverse	TCT ATT GGA TCC TTA TTT CCC GCT GGC GGT	*BamH*I
BLEF2	Forward	TCT CGC GGA TCC ATG CGT CGT CAT TCA TTT	*BamH*I
BLER2	Reverse	TCT ATT AAG CTT TTA TTT CCC GCT GGC GGT CAG	*Hind*III
BLMLF	Forward	CGC CGG CAT ATG GCT TCC CAC AAT CCG GTC	*Nde*I
