# Supplementary material for: Characterization of a novel subfamily 1.4 lipase from Bacillus licheniformis IBRL-CHS2: Cloning and expression optimization
Source: PLoS One. 2024 Dec 17;19(12):e0314556. doi: 10.1371/journal.pone.0314556 (PMC11651597; doi:10.1371/journal.pone.0314556)
Supplement: S2 Table — (PDF) [file pone.0314556.s002.pdf]

S2 Table: Components of natural oils used in this study

| Natural oils | Fatty acids (%) Saturated | Monounsaturated | Polyunsaturated |
|---|---|---|---|
| [1]Canola oil | 7.4 | 63.2 | 28.1 |
| [2]Olive oil | 14.5 | 75.7 | 9.8 |
| [2]Palm Oil | 47.7 | 41.9 | 10.4 |
| [2]Coconut oil | 92.4 | 6.2 | 1.4 |

1. https://en.wikipedia.org/wiki/Vegetable_oil
2. Gopala Krishna, A. G., Raj, G., Bhatnagar, A. S., Prasanth Kumar, P. K. and Chandrasekhar, P. (2010).

   Coconut oil: Chemistry, production and its application- A review. *Indian Coconut Journal*, 15-27.

| **Natural oils** | **Fatty acids (%)** Caprylic (C8) | Capric (C10) | lauric (C12) | Myristic (C14) | Palmitic (C16) | Stearic (C18) | Oleic (C18:1) | Linoleic (C18:2) | α-Linolenic (C18:3) |
|---|---|---|---|---|---|---|---|---|---|
| **[1]Canola** | - | - | - | - | 4 | 2 | 62 | 22 | 10 |
| **[2]Olive** | - | - | - | - | 12 | 2.5 | 75.7 | 7.9 | 0.5 |
| **[2]Palm** | - | - | 0.2 | 1.1 | 42.6 | 3.8 | 41.9 | 10.4 | - |
| **[2]Coconut** | 7 | 5.4 | 48.9 | 20.2 | 8.4 | 2.5 | 6.2 | 1.4 | - |

1: www.scientificpsychic.com/fitness/fattyacids.html

2: Gopala Krishna, A. G., Raj, G., Bhatnagar, A. S., Prasanth Kumar, P. K. and Chandrasekhar, P. (2010). Coconut oil: Chemistry, production and its application- A review. *Indian Coconut Journal*, 15-27.
