# Supplementary material for: Characterization of a novel subfamily 1.4 lipase from Bacillus licheniformis IBRL-CHS2: Cloning and expression optimization
Source: PLoS One. 2024 Dec 17;19(12):e0314556. doi: 10.1371/journal.pone.0314556 (PMC11651597; doi:10.1371/journal.pone.0314556)
Supplement: S1 Fig — The signal peptide was predicted to be the first 30 residues LipBL and the cleavage site is between Ala-30 and Ala-31. (PDF) [file pone.0314556.s003.pdf]

S1 Fig. Prediction of signal peptide in LipBL by SignalP server. The signal peptide was predicted to be the first 30 residues LipBL and the cleavage site is between Ala-30 and Ala-31

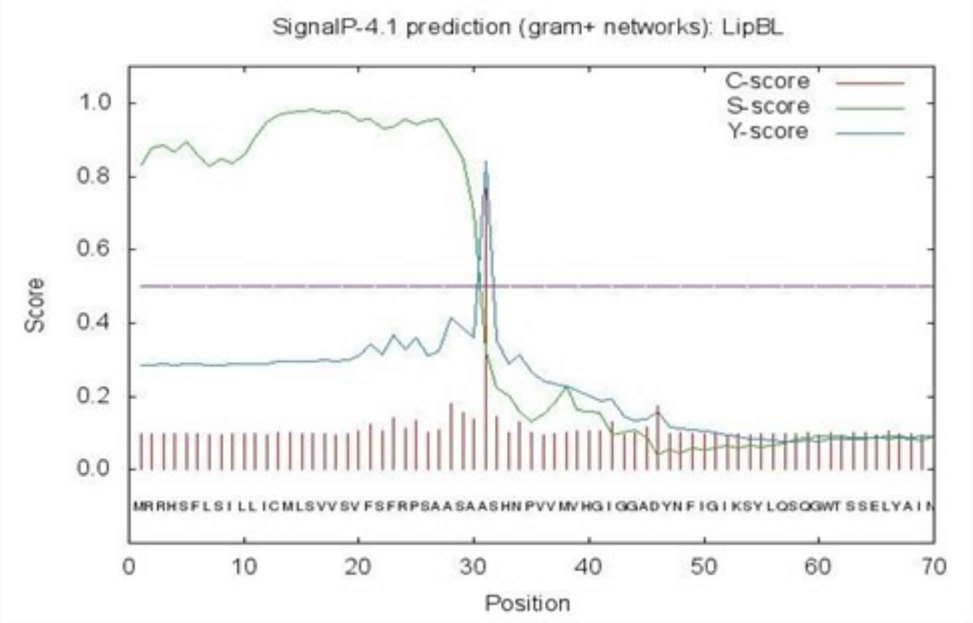


```
# Measure  Position  Value    Cutoff   signal peptide?
  max. C    31       0.765
  max. Y    31       0.840
  max. S    16       0.982
  mean S     1-30    0.910
       D     1-30    0.867   0.450    YES
Name=LipBL     SP='YES' Cleavage site between pos. 30 and 31: ASA-AS D=0.867 D-cutoff=0.450 Networks=SignalP-TM
```
