# Supplementary material for: Characterization of a novel subfamily 1.4 lipase from Bacillus licheniformis IBRL-CHS2: Cloning and expression optimization
Source: PLoS One. 2024 Dec 17;19(12):e0314556. doi: 10.1371/journal.pone.0314556 (PMC11651597; doi:10.1371/journal.pone.0314556)
Supplement: S2 Fig — (A) the genomic DNA extracted from Bacillus licheniformis IBRL-CHS2. The Lambda DNA/HindIII marker (Promega) is denoted as LM. The Genomic DNA is indicated by a white arrow in Lane 1. An agarose gel percentage of 0.7% was utilized for this analysis. (B, C) results of gel electrophoresis showing PCR products amplified with BLF and BLR (B) and the purified LipA gene, identified by the arrow, which is 615 bp in size (C). The samples were run on a 1% agarose gel for electrophoresis. (D) results of colony PCR products from 5 chosen white clones. The LipA gene, measuring 615 bp, is indicated by the arrow. (E) results of agarose gel electrophoresis (1%) following the EcoRI digestion of pGEM-LipA. Lane 1 shows the undigested pGEM- LipA in various conformations, resulting in bands of different sizes. The linear and monomer pGEM- LipA band is highlighted and labeled. In lane 2, digested pGEM- LipA shows the successful cleavage of the LipA gene from the pGEM-T Easy plasmid. (PDF) [file pone.0314556.s004.pdf]

S2 Fig. Displayed all the result of agarose gel electrophoresis using GeneRuler™ 1kb DNA ladder (Fermentas) which denoted as M. (A) the genomic DNA extracted from *Bacillus licheniformis* IBRL-CHS2. The Lambda DNA/*Hind*III marker (Promega) is denoted as LM. The Genomic DNA is indicated by a white arrow in Lane 1. An agarose gel percentage of 0.7% was utilized for this analysis. (B, C) results of gel electrophoresis showing PCR products amplified with BLF and BLR (B) and the purified LipA$_{B.licheniformis}$ gene, identified by the arrow, which is 615 bp in size (C). The samples were run on a 1% agarose gel for electrophoresis. (D) results of colony PCR products from 5 chosen white clones. The LipA$_{B.licheniformis}$ gene, measuring 615 bp, is indicated by the arrow. (E) results of agarose gel electrophoresis (1%) following the *EcoR*I digestion of pGEM-LipA$_{B.licheniformis}$. Lane 1 shows the undigested pGEM-LipA$_{B.licheniformis}$ in various conformations, resulting in bands of different sizes. The linear and monomer pGEM- LipA$_{B.licheniformis}$ band is highlighted and labeled. In lane 2, digested pGEM- LipA$_{B.licheniformis}$ shows the successful cleavage of the LipA$_{B.licheniformis}$ gene from the pGEM-T Easy plasmid.

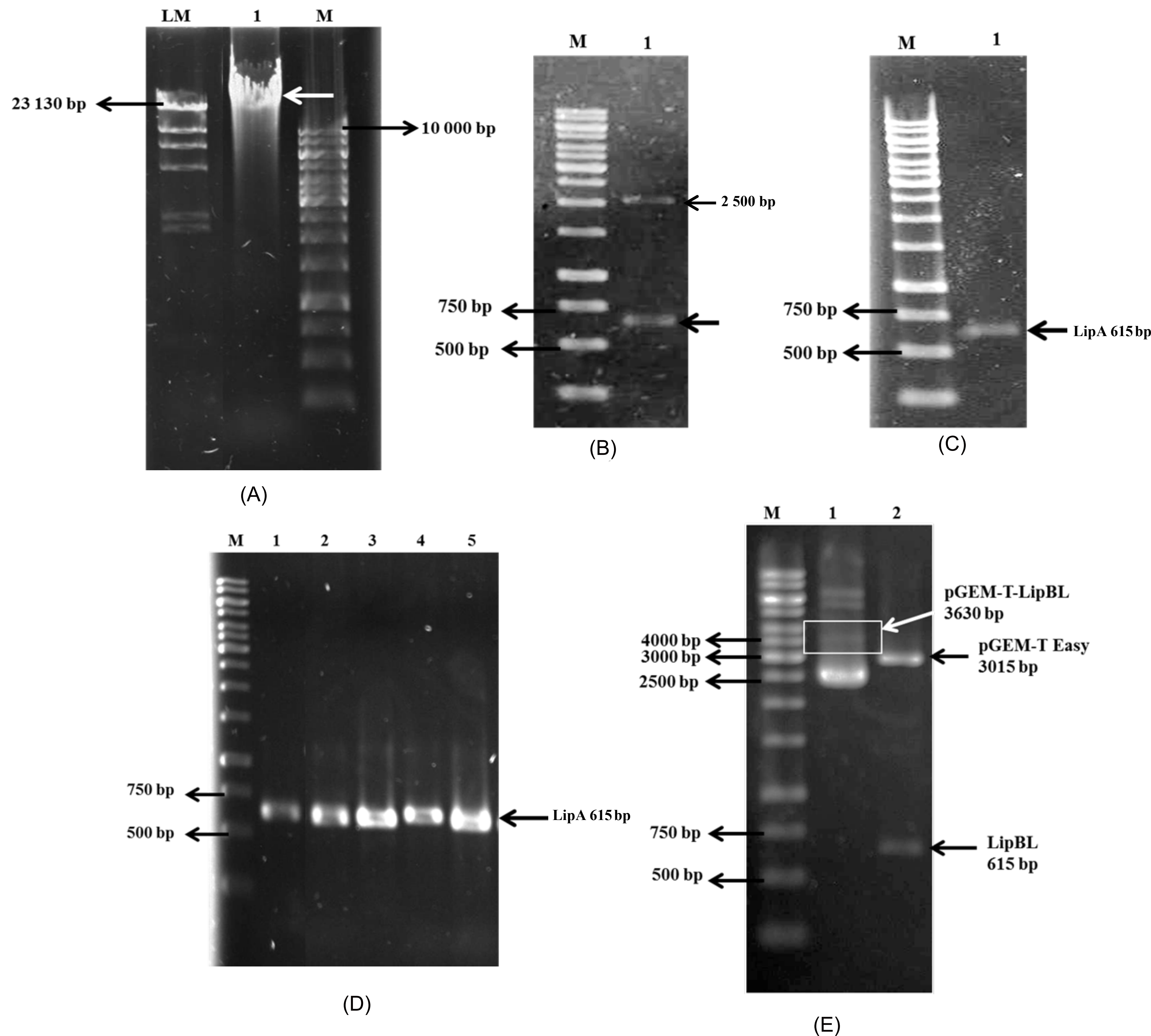
LM
1
M
23 130 bp
10 000 bp
(A)
M
1
2 500 bp
750 bp
500 bp
(B)
M
1
750 bp
500 bp
LipA 615 bp
(C)
M
1
2
3
4
5
750 bp
500 bp
LipA 615 bp
(D)
M
1
2
pGEM-T-LipBL
3630 bp
4000 bp
3000 bp
2500 bp
pGEM-T Easy
3015 bp
750 bp
500 bp
LipBL
615 bp
(E)
