# Supplementary material for: Characterization of a novel subfamily 1.4 lipase from Bacillus licheniformis IBRL-CHS2: Cloning and expression optimization
Source: PLoS One. 2024 Dec 17;19(12):e0314556. doi: 10.1371/journal.pone.0314556 (PMC11651597; doi:10.1371/journal.pone.0314556)

S3 Fig. displays a Lineweaver plot illustrating the relationship between substrate concentrations and enzyme activity of MLipA$_{B.licheniformis}$.

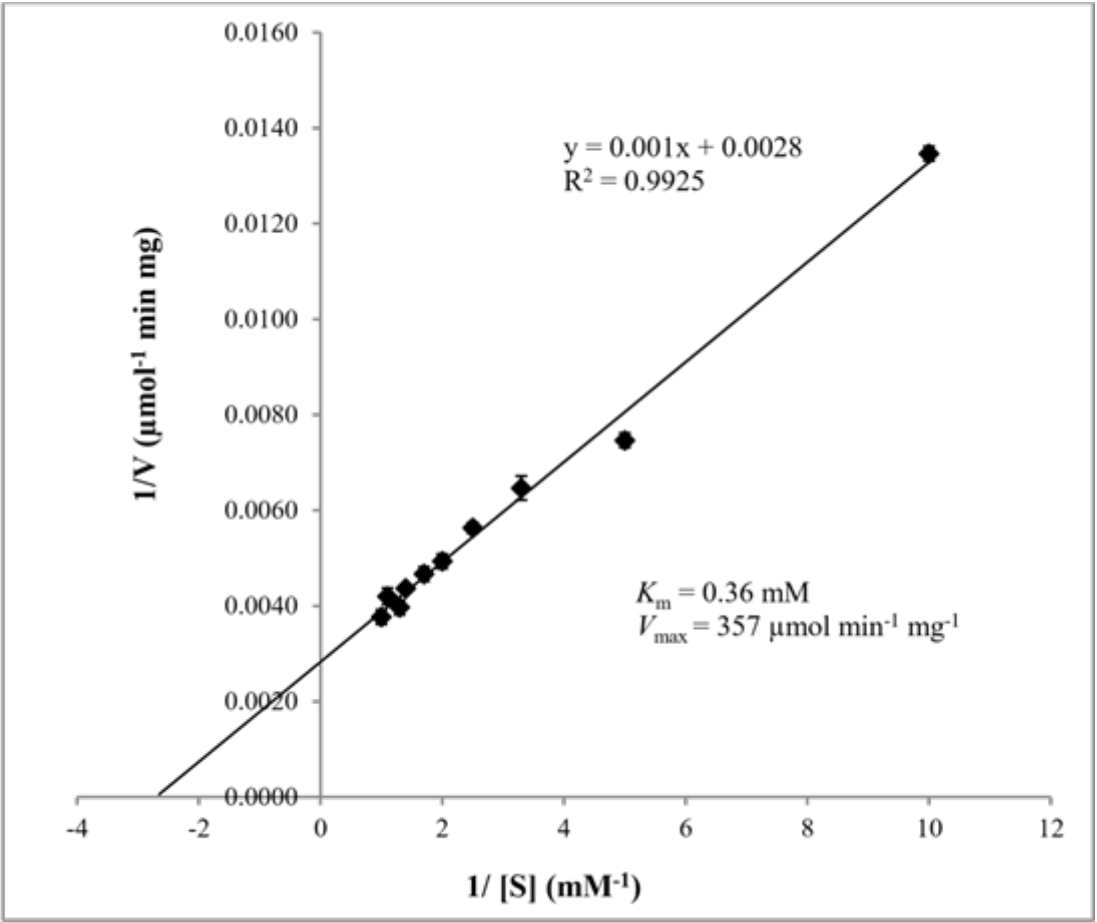

Supplement: S3 Fig — (PDF) [file pone.0314556.s005.pdf]
