# Supplementary material for: Characterization of a novel subfamily 1.4 lipase from Bacillus licheniformis IBRL-CHS2: Cloning and expression optimization
Source: PLoS One. 2024 Dec 17;19(12):e0314556. doi: 10.1371/journal.pone.0314556 (PMC11651597; doi:10.1371/journal.pone.0314556)
Supplement: S1 File — (PDF) [file pone.0314556.s006.pdf]

1. Lipase activity (U/mL):

$$\text{Lipase activity (U/mL)} = \frac{\text{amount of } p \text{ −nitrophenol released μmol /time of incubation (min)}}{\text{volume of lipase sample (mL)}}$$

2. Relative activity (%):

$$\text{Relative activity (\%)} = \frac{\text{Lipase activity at different temperature}}{\text{Lipase activity at optimum temperature (35°C)}} \times 100\%$$

3. Residual activity (%):

$$\text{Residual activity (\%)} = \frac{\text{Lipase activity at incubated temperature}}{\text{Lipase activity at control temperature (35 °C) without incubation}} \times 100\%$$
