# Supplementary material for: Characterization of a novel subfamily 1.4 lipase from Bacillus licheniformis IBRL-CHS2: Cloning and expression optimization
Source: PLoS One. 2024 Dec 17;19(12):e0314556. doi: 10.1371/journal.pone.0314556 (PMC11651597; doi:10.1371/journal.pone.0314556)
Supplement: S1 Raw images — (PDF) [file pone.0314556.s007.pdf]

Fig. 2. Displayed all the result of SDS-PAGE analysis with Lane M as BenchMark^{TM} Protein Ladder and Lane NC is induced *E. coli* BL21 (DE3) harbouring empty pCold (A,B) MLipA$_{B.licheniformis}$ expression in *E. coli* BL21 (DE3) harbouring pCold-MLipA$_{B.licheniformis}$ (A) and solubility of the expressed lipase (B); Lane UI is uninduced *E. coli* BL21 (DE3) harbouring pCold- MLipA$_{B.licheniformis}$; Lane 1 is crude cell extract; Lane 2 is soluble proteins; Lane 3 is inclusion bodies. (C) Expression of MLipA$_{B.licheniformis}$ in *E. coli* BL21 (DE3) harbouring pCold-MLipA$_{B.licheniformis}$ after induction with different IPTG concentrations at 15ºC. Lane1 is induction with 0 mM IPTG; Lane 2 is induction with 0.2 mM IPTG; Lane 3 is induction with 0.4 mM IPTG; Lane 4 is induction with 0.6 mM IPTG; Lane 5 is induction with 0.8 mM IPTG; Lane 6 is induction with 1.0 mM IPTG. (D) Time course analysis of the expression of MLipA$_{B.licheniformis}$ in *E. coli* BL21 (DE3) harbouring pCold- MLipA$_{B.licheniformis}$. Lane 1 is expression at 0 hour; Lane 2 is expression after 4 hours; Lane 3 is expression after 8 hours; Lane 4 is expression after 12 hours; Lane 5 is expression after 16 hours; Lane 6 is expression after 20 hours; Lane 7 is expression after 24 hours; Lane 8 is expression after 28 hours; Lane 9 is expression after 32 hours. (E) Purification of recombinant MLipA$_{B.licheniformis}$ by IMAC technique. Lane 1 is the diluted soluble crude lysate; Lane 2 is unbound proteins; Lanes 3-10 are wash fractions; Lanes 11-15 are eluate fractions containing purified lipase MLipA$_{B.licheniformis}$.

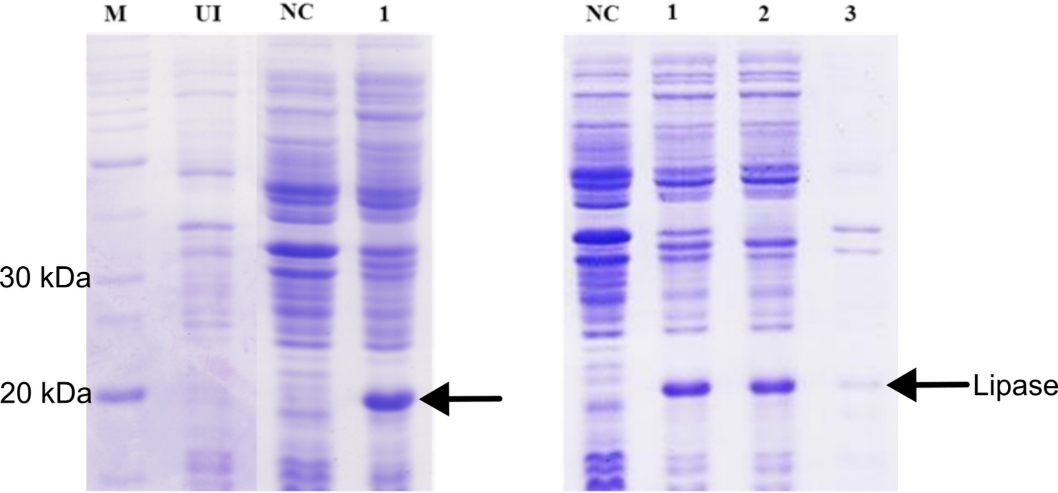

(A)
(B)

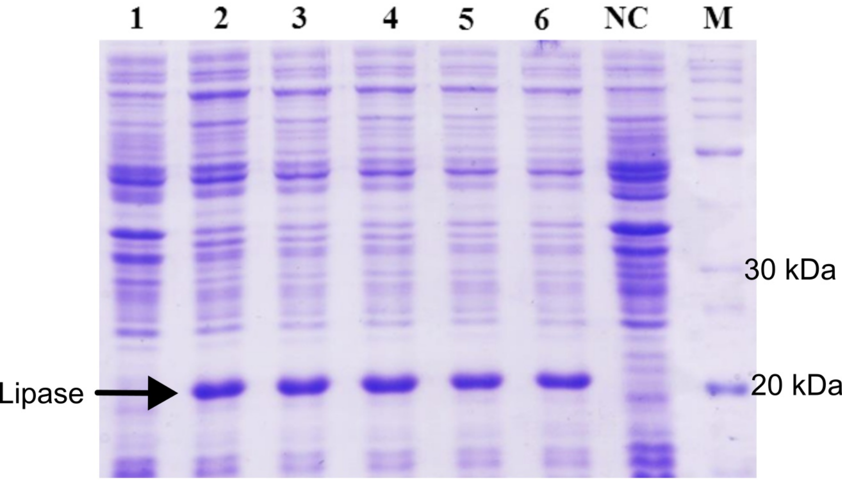

(C)

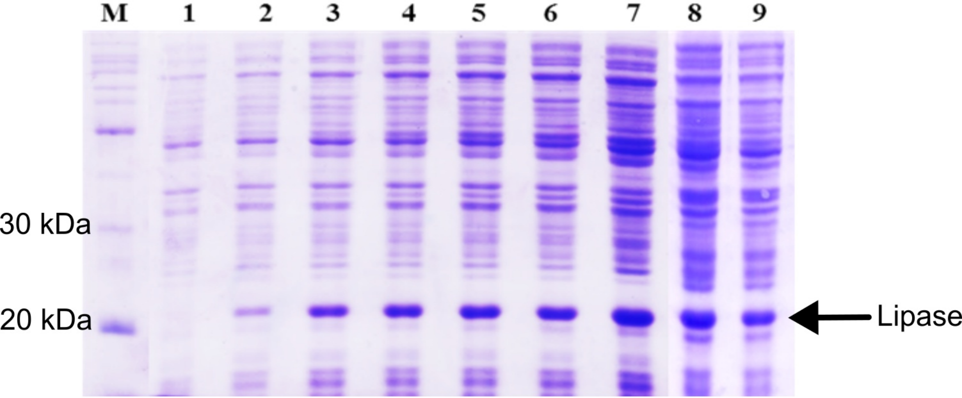

(D)

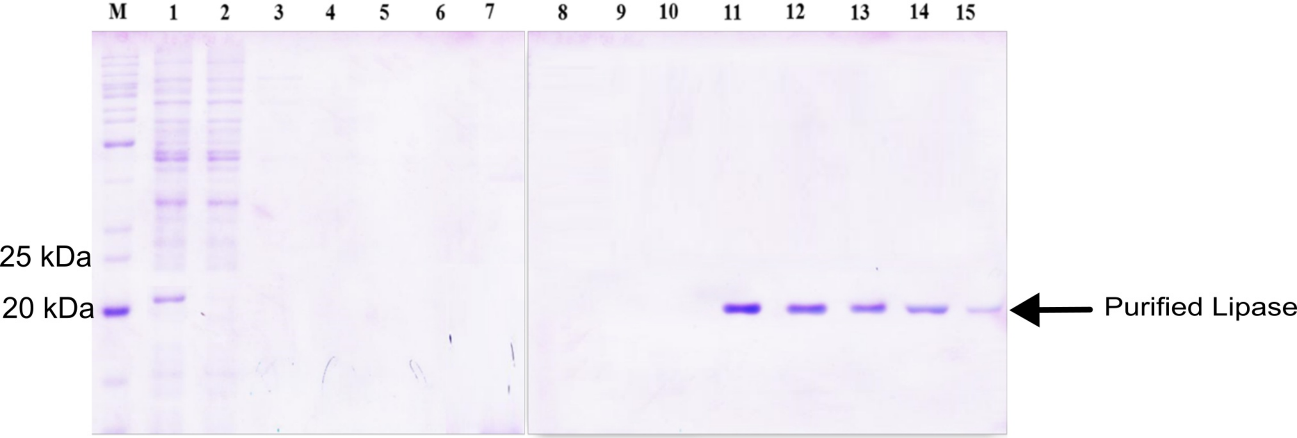

(E)

S2 Fig. Displayed all the result of agarose gel electrophoresis using GeneRuler^{TM} 1kb DNA ladder (Fermentas) which denoted as M. (A) the genomic DNA extracted from *Bacillus licheniformis* IBRL-CHS2. The Lambda DNA/*Hind*III marker (Promega) is denoted as LM. The Genomic DNA is indicated by a white arrow in Lane 1. An agarose gel percentage of 0.7% was utilized for this analysis. (B, C) results of gel electrophoresis showing PCR products amplified with BLF and BLR (B) and the purified LipA$_{B.licheniformis}$ gene, identified by the arrow, which is 615 bp in size (C). The samples were run on a 1% agarose gel for electrophoresis. (D) results of colony PCR products from 5 chosen white clones. The LipA$_{B.licheniformis}$ gene, measuring 615 bp, is indicated by the arrow. (E) results of agarose gel electrophoresis (1%) following the *EcoR*I digestion of pGEM-LipA$_{B.licheniformis}$. Lane 1 shows the undigested pGEM-LipA$_{B.licheniformis}$ in various conformations, resulting in bands of different sizes. The linear and monomer pGEM- LipA$_{B.licheniformis}$ band is highlighted and labeled. In lane 2, digested pGEM- LipA$_{B.licheniformis}$ shows the successful cleavage of the LipA$_{B.licheniformis}$ gene from the pGEM-T Easy plasmid.

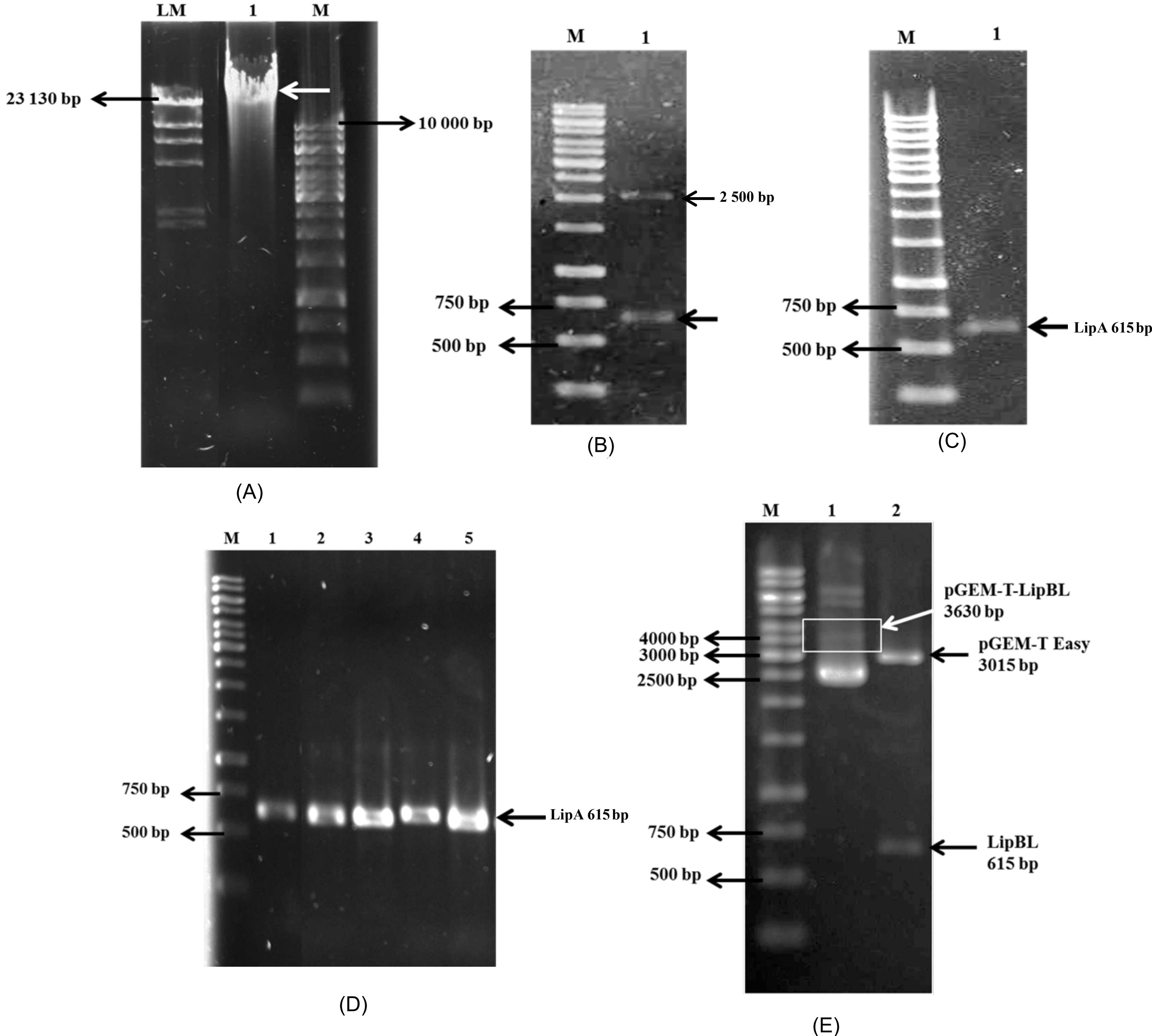
LM
1
M
23 130 bp
10 000 bp
(A)
M
1
2 500 bp
750 bp
500 bp
(B)
M
1
750 bp
LipA 615 bp
500 bp
(C)
M
1
2
3
4
5
750 bp
500 bp
LipA 615 bp
(D)
M
1
2
pGEM-T-LipBL
3630 bp
4000 bp
3000 bp
2500 bp
pGEM-T Easy
3015 bp
750 bp
500 bp
LipBL
615 bp
(E)
