# Supplementary material for: Characterization of a novel subfamily 1.4 lipase from Bacillus licheniformis IBRL-CHS2: Cloning and expression optimization
Source: PLoS One. 2024 Dec 17;19(12):e0314556. doi: 10.1371/journal.pone.0314556 (PMC11651597; doi:10.1371/journal.pone.0314556)
Supplement: Attachment — Submitted filename: Response to reviewers.docx [file pone.0314556.s008.docx]

**Responses to reviewers**

We sincerely thank the reviewers for their valuable comments and suggestions, which have greatly contributed to the improvement of our manuscript. Below, we have addressed each point in detail.

**Table 1: Main Comments and Responses**

| Main Comment | Response | Location |
| --- | --- | --- |
| What is the originality of the study? Please mention in detail. | Thank you for your valuable comment. The originality of this study has been updated in the manuscript. | lines 107–133. |
| The species name in the enzyme abbreviation should be removed. | Thank you for your valuable comment. The species name has been appropriately removed from all enzyme abbreviations throughout the manuscript for clarity and consistency. | Whole text |
| Enzyme assay should be detailed. | Thank you for your comment. The enzyme assay has been expanded to include detailed procedures, ensuring a comprehensive understanding of the experimental conditions and methodology used. | Lines 162-183. |
| Statistical analysis should be added. | Thank you for your comment regarding statistical analysis. I have added a "Statistical Analysis" section to the manuscript. All experiments were performed in triplicate, and the results are presented as mean ± standard deviation (SD). R-values were also calculated to ensure the linearity and reliability of the experimental data. | Lines 270-275. |

**Table 2: Specific Comments and Responses**

| Specific Comment | Response | Location |
| --- | --- | --- |
| The title should be improved to be more attractive and concise. | Thank you for your valuable comment. The title has been updated. | Lines 3-4. |
| The first sentence in the Abstract should be removed or moved to the introduction section since there are no provided results. | Thank you for your comment. The first sentence has been removed from the Abstract to improve the flow of information. | - |
| The acronyms “DMSO”, “SDS”, “PAGE”, “PET”, “OD”, “IPTG”, etc. should be spelled out. | Thank you for your comment. All abbreviations such as DMSO, SDS, PET, OD, IPTG have been spelled out at their first mention to enhance clarity and ensure consistency throughout the manuscript. | Lines 38, 42, 48 and 102. |
| Please correctly provide the expression unit of the Vmax where “+1” in “min” and “mg” are in superscript. | Thank you for your comment. The expression units for V_max_ have been corrected, with the appropriate superscripting of "+1" in “min” and “mg,” ensuring that the units are properly formatted. | Line 44 |
| Keywords: Please remove “LipAB.licheniformis” and “MLipAB.licheniformis (Mature lipase)”. | The keywords “LipAB.licheniformis” and “MLipAB.licheniformis (Mature lipase)” have been removed as per the reviewer’s suggestion to streamline the list of keywords. | Lines 50-51 |
| Introduction section should be enhanced and needs revision to properly address the focus of the study. | Thank you for your valuable comment. The Introduction has been significantly revised to better align with the focus of the study, providing a clearer and more concise context for the research. | Lines 107-133. |
| The authors are invited to provide updated information about the classification of lipases from LIPABASE. | Thank you for your valuable comment. Updated information regarding lipase classification has been added from the LIPABASE database, enriching the background and providing a more comprehensive overview of lipase family enzymes. | Lines 72-81. |
| The authors are invited to provide the novelty of their work compared to the study reported by Reddy et al., (2016). | Thank you for your comment. The novelty of our work, in comparison to the study by Reddy et al. (2016), has been addressed and updated in the manuscript. Please refer to Lines (Lines 108-132) where we have detailed how our study overcomes the limitations of the previous work and highlights the unique properties of the enzyme we characterized. | Lines 118-132. |
| Please correct as “min” instead of “mins” or “minutes”, and “h” instead of “hours” or “hrs”. | Thank you for your comment. All instances of “mins” and “hours” have been corrected to “min” and “h” to ensure consistency with standard scientific notation. | Whole text |
| The word “Stability” is missing in the sub-title and “H” is missing after “Tris-”. | Thank you for your comment. The sub-title has been updated to include the word "Stability," and "H" has been added after “Tris-” to accurately reflect the buffer composition. | Lines 192, 193 and 199. |
| The personal word “we” should be replaced by another expression. | Thank you for your comment. All personal expressions using "we" have been replaced with more objective phrasing to adhere to formal scientific writing standards. | Whole text |
| Be sure to consistently list manufacturer locations. | Thank you for your feedback. I have ensured that all manufacturer locations are consistently listed throughout the manuscript. | Whole text |
| The authors said that “The control reaction, which did not contain any metal ions, was also prepared. The lipase activity in the presence of each metal ion was compared to the control reaction to calculate the relative activity (%).” To be convinced, the author should mention that the control for the metal cations MUST be measured after dialyzed against used buffer and/or measured in the presence of strong metallic ion chelators (for example 10 mM EDTA or 1 mM EGTA). | Thank you for your insightful comment. We have revised the manuscript to clarify the preparation of the control reaction. Specifically, in the **Methods** section, we now explicitly state that the lipase sample for the control reaction was dialyzed against the buffer used in the experiment to eliminate any trace metal ions that could influence the activity. This ensures that the control is free from any unintended metal ion contamination and allows for accurate comparison with the metal-ion treated samples.  We also acknowledge your suggestion regarding the inclusion of strong metal chelators, such as 10 mM EDTA or 1 mM EGTA, in the control setup. Although chelators were not included in this particular study, we agree that their use in future experiments would further ensure the complete removal of any potential trace metal ions, providing even more robust results. We will incorporate this modification in future studies to validate the observations further. | Lines 246-250. |
| Errors in citation presentation (e.g., L212). | Thank you for your comment. Citation formatting errors, such as “L212,” have been corrected to ensure proper citation style throughout the manuscript. | Line 267 |
| The name of restriction enzymes should not be in italics. | Thank you for your comment. The italicization of restriction enzyme names has been removed in accordance with IUPAC guidelines, ensuring correct nomenclature. | Whole text |
| Table 1: How many times were the experiments conducted? Provide standard error (±SD) values. | Thank you for your comment regarding Table 1. I have revised the table to include the standard deviation (±SD) values, as requested. The purification experiments were conducted in triplicate, and the updated table now reflects the appropriate means and SD values for each parameter. | Lines 350-352 |
| L319 and L582: Please correctly provide the name of “MLipAB.licheniformis's”. | Thank you for your feedback. The name “MLipAB.licheniformis's” has been corrected to align with proper scientific terminology. | Whole text |
| L501: Please correct as “Strain ARM”. | Thank you for your comment. The term “strain ARM” has been corrected for clarity and accuracy. | Line 551 |
| Please remove the oldest references if not necessary. | Thank you for your feedback. The older references were selected because they focus specifically on *Bacillus* lipases, which are directly relevant to my study. There are limited new references specifically on *Bacillus* lipases, and the newer studies often involve lipases from different sources. To maintain a logical and meaningful comparison, I prioritized older studies on *Bacillus* lipases, which align more closely with my research focus. | - |
| Please re-check and consult the updated guides to ensure compliance with journal style. | Thank you for your feedback. I have reviewed the manuscript in accordance with the updated guidelines to ensure compliance with the journal’s style. | Whole text |
| Ref no.: 76: Provide the correct journal abbreviation in accordance with Index Medicus and Web of Science. | Thank you for your feedback. The corrected journal abbreviation is verified per Index Medicus and Web of Science. | Line 1134 |
| Some spelling and grammar mistakes should be corrected. | Thank you for your feedback. I have carefully reviewed the manuscript and corrected the spelling and grammar mistakes as suggested. | Whole text |

**Data availability response**

Thank you for your comments regarding the Data Availability Statement. The manuscript contains all the essential data needed to replicate the findings of this study. However, due to ongoing enhancements, including the application of peptides to improve lipase stability, some original data is not yet fully formatted for public sharing.

We plan to share all relevant data in future publications once the enhancements are complete. In the meantime, we kindly request that the data provided within the manuscript be considered sufficient to replicate the current study’s results. Should the editor or any reviewers have specific questions or require access to the data during the review process, we are happy to provide these privately upon request.
